# To Extirpate House Insects

**Published:** 1876-07

**Authors:** 


### To Extirpate House Insects.

We have alluded to the usefulness of alum for this purpose in a former number, and now the *Journal of Chemistry* publishes a recipe for the destruction of insects, which is a slight modification of what we have advised, and maybe more. Take two pounds of alum and dissolve it in three or four quarts of boiling water; let it stand on the fire till the alum disappears; then apply it with a brush, while nearly boiling hot, to every joint and crevice in your closets, bedsteads, pantry shelves, and the like. Brush crevices in the floor of the skirting, or mop boards, if you suspect they harbor vermin.—*Phren. Journal.*

